# A Gene Signature of Survival Prediction for Kidney Renal Cell Carcinoma by Multi-Omic Data Analysis

**DOI:** 10.3390/ijms20225720

**Published:** 2019-11-14

**Authors:** Fuyan Hu, Wenying Zeng, Xiaoping Liu

**Affiliations:** 1 Department of Statistics, Faculty of Science, Wuhan University of Technology, 122 Luoshi Road, Wuhan 430070, China; fuyanhu@whut.edu.cn; 2 Department of Water Resources and Hydro-elctricity Engineering, College of Water Resources and Architectural Engineering, Northwest A&F University, No.3 Taicheng Road, Yangling 712100, China; zwyxn016@163.com; 3 School of Mathematics and Statistics, Shandong University at Weihai, Weihai 264209, China

**Keywords:** kidney renal cell carcinoma, multi-omics analysis, prognosis signatures, LASSO Cox regression, multivariate stepwise analysis

## Abstract

Kidney renal cell carcinoma (KIRC), which is the most common subtype of kidney cancer, has a poor prognosis and a high mortality rate. In this study, a multi-omics analysis is performed to build a multi-gene prognosis signature for KIRC. A combination of a DNA methylation analysis and a gene expression data analysis revealed 863 methylated differentially expressed genes (MDEGs). Seven MDEGs (*BID*, *CCNF*, *DLX4*, *FAM72D*, *PYCR1*, *RUNX1*, and *TRIP13*) were further screened using LASSO Cox regression and integrated into a prognostic risk score model. Then, KIRC patients were divided into high- and low-risk groups. A univariate cox regression analysis revealed a significant association between the high-risk group and a poor prognosis. The time-dependent receiver operating characteristic (ROC) curve shows that the risk group performs well in predicting overall survival. Furthermore, the risk group is contained in the best multivariate model that was obtained by a multivariate stepwise analysis, which further confirms that the risk group can be used as a potential prognostic biomarker. In addition, a nomogram was established for the best multivariate model and shown to perform well in predicting the survival of KIRC patients. In summary, a seven-MDEG signature is a powerful prognosis factor for KIRC patients and may provide useful suggestions for their personalized therapy.

## 1. Introduction

Kidney cancer is a complicated disease that is composed of a variety of different types of kidney tumors. Renal cell carcinoma (RCC) accounts for 90% of all kidney cancers. The main three types of kidney cancer are: kidney renal clear cell carcinoma or clear cell RCC (KIRC or ccRCC, accounting for 70–75% of all kidney cancers), kidney renal papillary cell carcinoma or papillary RCC (KIRP or pRCC, accounting for 10–16% of all kidney cancers), and kidney chromophobe or chromophobe RCC (KICH or chRCC, accounting for 5% of all kidney cancers) [1]. KIRC, as the most common subtype of kidney cancer, usually has the worst overall survival rate [2]. The loss of the von Hippel–Lindau (*VHL*) gene was found to be involved in more than 60% of all sporadic renal cell cancers, which results in a high expression level of hypoxia-inducible factors (HIFs) and their downstream genes, including vascular endothelial growth factor (*VEGF*) [3]. Studies suggest that VHL-HIF is a key oncogenesis mechanism in KIRC [4]. According to this mechanism, tyrosine kinase inhibitors (TKIs), including sunitinib and pazopanib, have been developed to treat KIRC patients by targeting the VEGF pathway, and have shown good performance in increasing patients’ overall survival rate under certain circumstances [5,6]. Besides *VHL*, frequent genetic mutations have been detected in *BAP1*, *PBRM1*, and *SETD2*, all of which are related to chromatin and histone regulation and serve as tumor suppressor genes in kidney cancer. Bihr et al. concluded that a combination of *PBRM1*, *BAP1*, *SETD2*, and *VHL* dysfunction is fatal to KIRC progression [7]. It was reported that *VHL* mutation has no prognostic value [8], while *BAP1* mutation was related to overall survival in KIRC patients [9]. The *SETD2* protein was reported to be a good prognosis marker in KIRC [10]. In RCC, decreased *PBRM1* expression is associated with a poor prognosis [11]. Considering mutation information may provide us with new insights into the prognosis of KIRC.

Although many prognosis factors have been reported for KIRC, the pathological stage of RCC patients remains the main predictor of prognosis [12]. However, survival differences exist between patients at the same stage [13]. Therefore, new reliable prognostic biomarkers urgently need to be developed in order to realize personalized medicine and treatment. In addition, DNA methylation is a major epigenetic modification that plays important roles in tumor development. Many genes (such as *CRB3* [14], *SFRP1* [15], *ZNF492*, and *GPR149* [16]) with an abnormal methylation status have been reported to be prognosis biomarkers for KIRC. Signatures formed by multiple genes with a changed methylation status have been reported to be powerful predictors of prognosis for hepatocellular carcinoma [17] and pancreatic cancer [18]. Recently, a nine-gene signature and a five-gene signature were reported to be prognosis biomarkers for KIRP and stage III KIRC, respectively [19,20]. However, both studies did not use the methylation information. Moreover, multi-omics based survival prediction models has been applied to cancers, such as breast cancer [21] and pancreatic ductal adenocarcinoma [22].

In our study, we obtained a seven-gene signature by combining methylation and expression profiles to provide a reliable prognostic model for KIRC patients.

## 2. Results

### 2.1. Summary of Datasets

**Figure 1:**
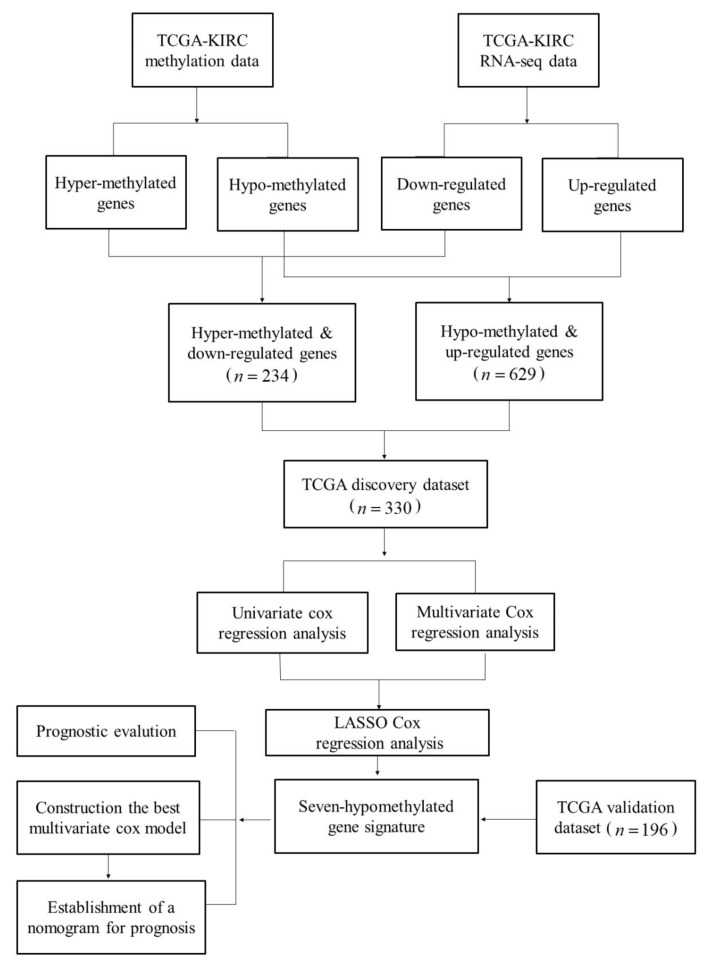
The pipeline of our method.

There were 529 KIRC samples and 79 normal samples with the Cancer Genome Atlas (TCGA, http://cancergenome.nih.gov/) expression data. There were 537 patients with clinical information. In addition, 336 samples had somatic mutation information. After comparing, we divided the TCGA data into two groups: 330 patients for whom three types of data were available (clinical data, a gene expression profile, and somatic mutation information) were used as a TCGA discovery dataset; and 196 patients for whom only clinical data and gene expression information were available were used as a TCGA validation dataset. Table 1 lists information on the TCGA discovery dataset and the validation dataset. Figure 1 shows the flowchart of this study.

The TCGA gene expression data contained 20,501 genes. We omitted 2398 genes that were not expressed in more than 50% of the samples, leaving 18,103 genes for the study.

DNA methylation data on 485,577 loci for paired KIRC samples and normal samples were obtained from the TCGA. After filtering the loci without a value for more than 50 samples, 396,064 loci remained for further analysis.

The somatic mutation data contained 26,693 somatic mutations involving 9290 genes. However, most genes had a very low mutation frequency. The somatic mutation information for the top five genes (*VHL*, *PBRM1*, *SETD2*, *TTN*, and *BAP1*) with the highest mutation frequency were extracted for this study (Table 1). In detail, *VHL* had the highest mutation frequency, with a mutation in 150 patients (accounting for 45.5% of all patients in the TCGA discovery dataset). *PBRM1* had the second-highest mutation frequency, with a mutation in 128 patients (accounting for 38.8% of all patients in the TCGA discovery dataset). *TTN* had the third-highest mutation frequency, with a mutation in 58 patients (accounting for 17.6% of all patients in the TCGA discovery dataset). *SETD2* had the fourth-highest mutation frequency, with a mutation in 39 patients (accounting for 11.8% of all patients in the TCGA discovery dataset). *BAP1* had the fifth-highest mutation frequency, with a mutation in 29 patients (accounting for 8.8% of all patients in the TCGA discovery dataset).

### 2.2. Identification and Enrichment Analysis of Methylated Differentially Expressed Genes (MDEGs) in Kidney Renal Cell Carcinoma (KIRC)

**Figure 2:**
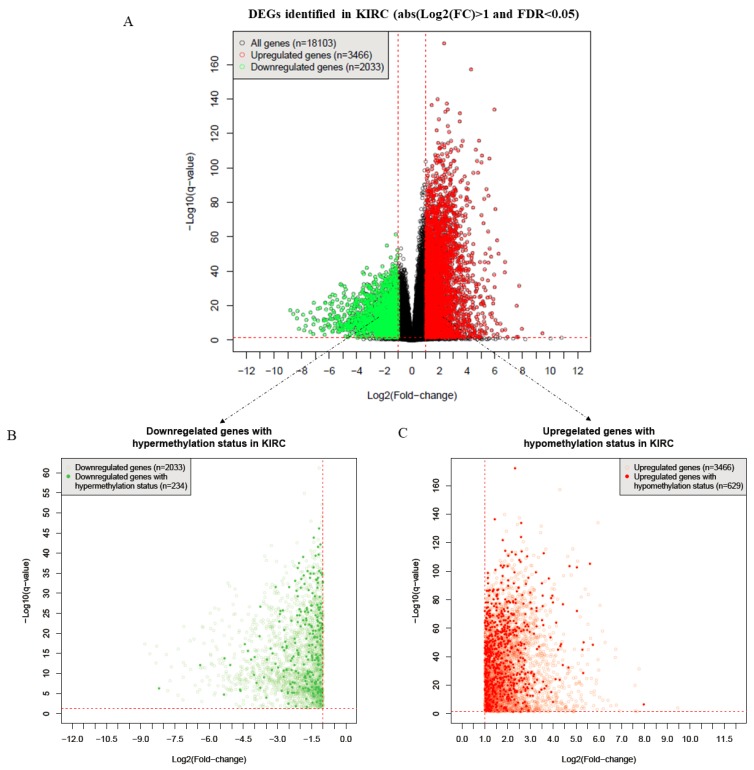
The process of selecting methylated differentially expressed genes (MDEGs). (**A**) The DEGs identified in kidney renal cell carcinoma (KIRC) (*n* = 5499). (**B**) Downregulated genes with hypermethylation status in KIRC (*n* = 234). (**C**) Upregulated genes with hypomethylation status in KIRC (*n* = 629).

After performing Student’s *t*-test, a Benjamini–Hochberg correction was conducted to obtain the false discovery rate (FDR, i.e., the *q*-value) [23]. Then, with cut-off values of FDR < 0.05 and $\left|\log_{2}(FC)\right|>1$, 2033 downregulated and 3466 upregulated genes were identified as being differentially expressed genes (DEGs) (Figure 2A). Furthermore, by comparison with the DNA methylation status, 863 DEGs were detected as being MDEGs in KIRC, including 234 downregulated genes with a hypermethylated status (Figure 2B) and 629 upregulated genes with a hypomethylated status (Figure 2C).

**Figure 3:**
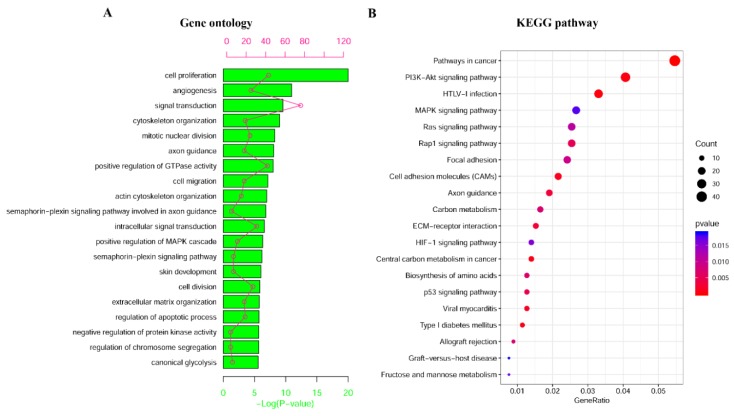
Enrichment analysis of MDEGs. (**A**) The top 20 significant enriched Gene Ontology (GO) for 863 MDEGs. (**B**) The top 20 significant enriched KEGG pathways for 863 MDEGs.

Moreover, a function enrichment analysis was done through the database for annotation, visualization, and integrated discovery (DAVID, https://david.ncifcrf.gov/) for the 863 MDEGs, which revealed that these MDEGs were significantly enriched in cell proliferation, angiogenesis, and signal transduction, among other things (Figure 3A). Enriched Kyoto Encyclopedia of Genes and Genomes (KEGG, https://www.genome.jp/kegg/) pathways were also detected for these MDEGs. The analysis showed that they were enriched in pathways in cancer, HTLV-I infection, and PI3K-Akt signaling, among others (Figure 3B). Therefore, MDEGs were found to be involved in many important biological processes and pathways in cancer.

### 2.3. Construction and Assessment of a Prognostic Risk score Model for KIRC

By performing both a univariate and a multivariate Cox regression analysis for the 863 MDEGs, we identified 189 MDEGs as potential prognosis-related genes (*p*-value < 0.05). Then, we performed LASSO Cox regression on the 189 MDEGs to select the most informative gene set for prognosis (Figure S1). Eventually, seven MDEGs (*BID*, *CCNF*, *DLX4*, *FAM72D*, *PYCR1*, *RUNX1*, and *TRIP13*) that were highly associated with prognosis were selected as features for a risk score model (Table 2). Finally, we constructed a formula to calculate the risk score for predicting prognosis based on the expression levels of the seven genes in KIRC patients.

(1) $$\begin{array}{ll} RS & =0.073*BID+0.062*CCNF+0.116*DLX4+0.061*FAM72D\\ & +0.026*PYCR1+0.107*RUNX1+0.058*TRIP13. \end{array}$$

where “*RS*” is short for “risk score”; the number before each gene symbol in Formula (1) is the regression coefficient of the gene in the best LASSO Cox regression model; and the gene symbol represents the expression of this gene.

**Figure 4:**
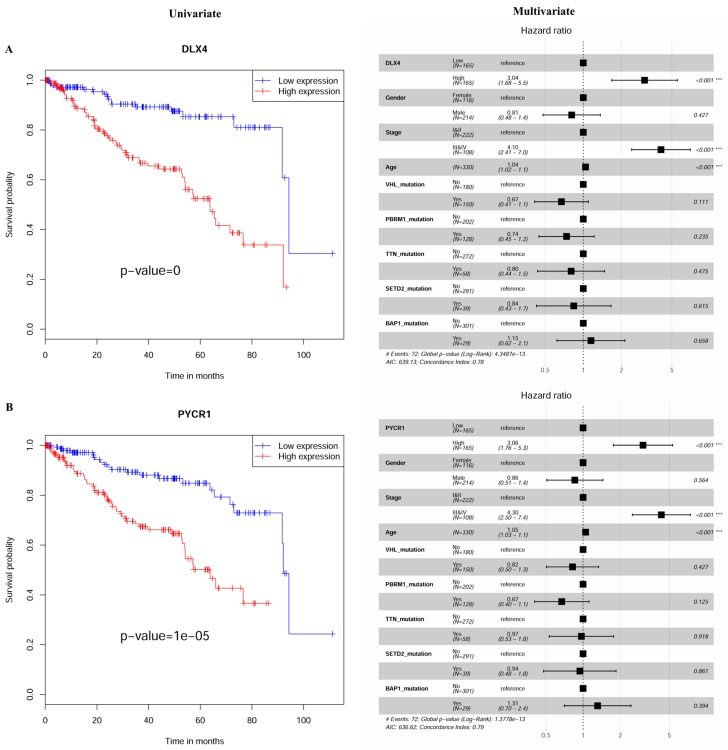
The expression signature that is associated with survival outcomes in KIRC. (**A**) Kaplan–Meier curves show the overall survival of KIRC patients with a high and a low expression level of *DLX4*. Patients with a high expression level of *DLX4* exhibited a significantly worse outcome compared with those with a low expression level of *DLX4*. A multivariable Cox regression analysis showed that *DLX4* was an independent prognostic factor as compared with other confounding factors. (**B**) *PYCR1* was found to be an independent prognostic marker for KIRC. A high expression level of this factor was associated with a worse outcome.

**Figure 5:**
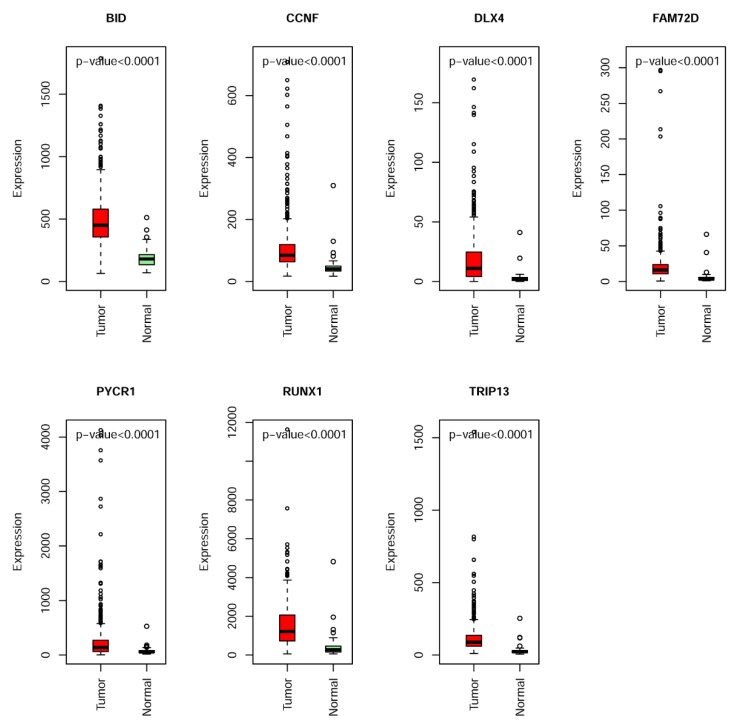
The expression level of seven hypomethylated genes (*BID*, *CCNF*, *DLX4*, *FAM72D*, *PYCR1*, *RUNX1*, and *TRIP13*) in KIRC and normal samples.

Figure 4 and Figures S2–S6 show the univariate and multivariate Cox regression analysis results (Table 2) for the selected seven MDEGs (*BID*, *CCNF*, *DLX4*, *FAM72D*, *PYCR1*, *RUNX1*, and *TRIP13*). The differential expression of the seven genes between tumor and normal samples suggests that they could be used as potential biomarkers for the diagnosis of KIRC patients (Figure 5).

**Figure 6:**
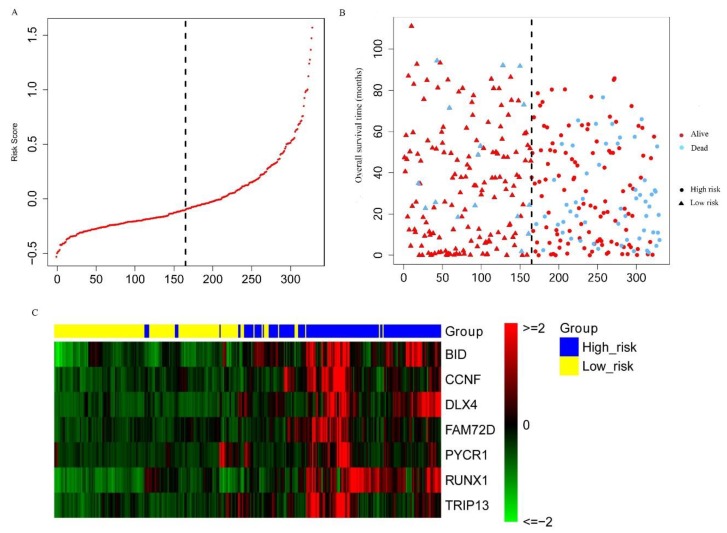
Construction of the seven-MDEG signature of KIRC. (**A**) The risk score distribution of KIRC patients. (**B**) The survival status of each patient. (**C**) The expression heatmap of the seven MDEGs in the discovery dataset (*n* = 330). Red: high expression; green: low expression.

Furthermore, according to the formula of the risk score model (Equation 1), a risk score can be calculated for each patient. The patients were divided into high- and low-risk subgroups by using the median of the risk scores as a cut-off. The distribution of the risk scores is shown in Figure 6A. The survival status for the high- and low-risk subgroups is shown in Figure 6B, which shows that the high-risk subgroup contained a higher number of dead patients than the low-risk subgroup. The expression heatmap for the seven MDEGs shows that they tend to have a higher expression level in the high-risk subgroup as compared with the low-risk subgroup (Figure 6C).

**Figure 7:**
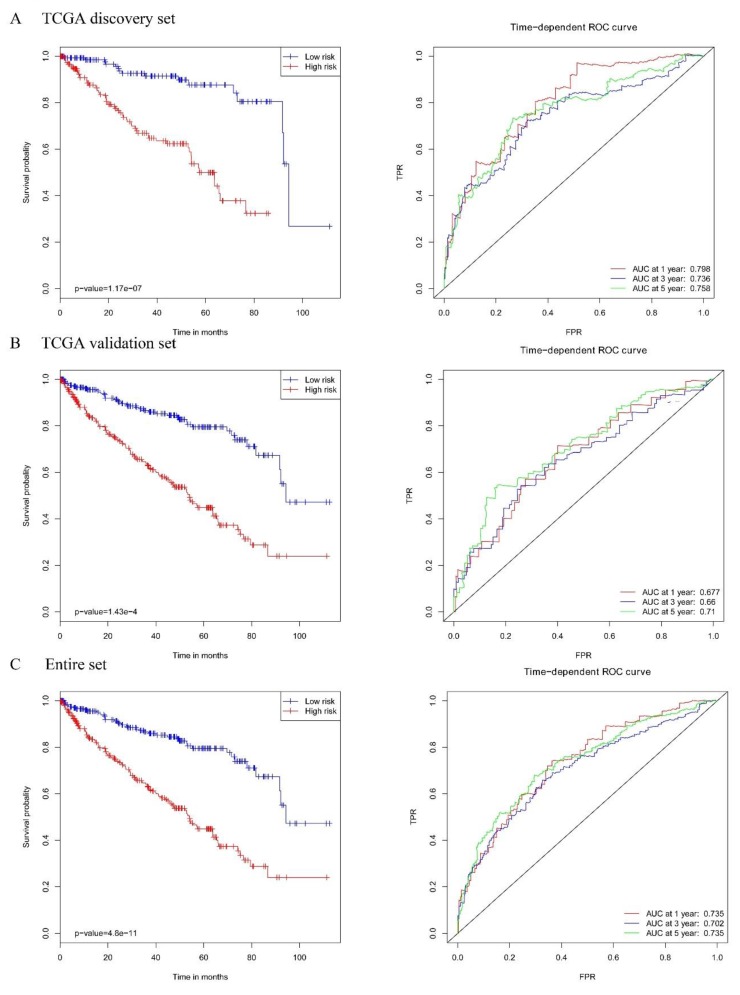
The Kaplan–Meier plot (low-risk vs. high-risk group) for KIRC patients (left panel); Receiver operating characteristic (ROC) analysis of the sensitivity and specificity of the survival time by a risk score based on the seven-MDEG signature of KIRC (right panel) in (**A**) the TCGA discovery dataset, (**B**) the TCGA validation set, and (**C**) the entire set.

Significant survival differences between the high-risk subgroup and the low-risk subgroup were observed in the TCGA discovery dataset (Figure 7A, left panel; the *p*-value of the log-rank is 1.17 × 10^−7^). The receiver operating characteristic (ROC) analysis showed that the risk score based on the seven MDEGs performed well in death prediction (Figure 7A, right panel). The area under the curve (AUC) at 1, 3, and 5 years was 0.798, 0.736, and 0.758, respectively, in the TCGA discovery dataset. To confirm the prognostic value of the risk score, we validated it using the TCGA validation dataset. Similar results were achieved in the validation dataset. Significant survival differences between the high-risk subgroup and the low-risk subgroup were also observed (Figure 7B, left panel; the *p*-value of the log-rank is 1.43 × 10^−4^). The AUC at 1, 3, and 5 years was 0.677, 0.66, and 0.71, respectively, in the TCGA validation dataset (Figure 7B, right panel). In the analysis of the entire set, we also obtained similar results. The high-risk subgroup had a worse survival rate (Figure 7C, left panel; the *p*-value of the log-rank is 4.80 × 10^−11^). The AUC at 1, 3, and 5 years was 0.735, 0.702, and 0.735, respectively, in the entire TCGA dataset (Figure 7C, right panel).

Univariate and multivariate Cox regression analyses were performed for the risk group (Table 3). In the univariate analysis, tumor stage (hazard ratio (HR) = 4.69, *p*-value = 1.48 × 10^−9^), age (HR = 1.04, *p*-value = 2.25 × 10^−5^), KIRC risk group based on the seven MDEGs (HR = 5.15, *p*-value = 1.17 × 10^−7^), and mutation status of *BAP1* (HR = 2.05, *p*-value = 0.01) were significantly associated with overall survival (OS) in KIRC patients. To compare the prognostic value of the risk group (high-risk subgroup and low-risk subgroup) to other prognostic confounders (Table 1), a multivariate stepwise analysis was performed. In the end, our recorded risk group (HR = 3.92, *p*-value = 2.25 × 10^−5^), tumor stage (HR = 3.47, *p*-value = 2.80 × 10^−6^), age (HR = 1.04, *p*-value = 3.62 × 10^−5^), and mutation status of *VHL* (HR = 0.68, *p*-value = 0.11) remained as independent prognostic biomarkers (Table 3). The best multivariate model included our recorded risk group, which further confirms that our recorded risk group can be used as a prognostic subgroup.

### 2.4. Establishment of a Nomogram for Overall Survival (OS) Prediction in KIRC

**Figure 8:**
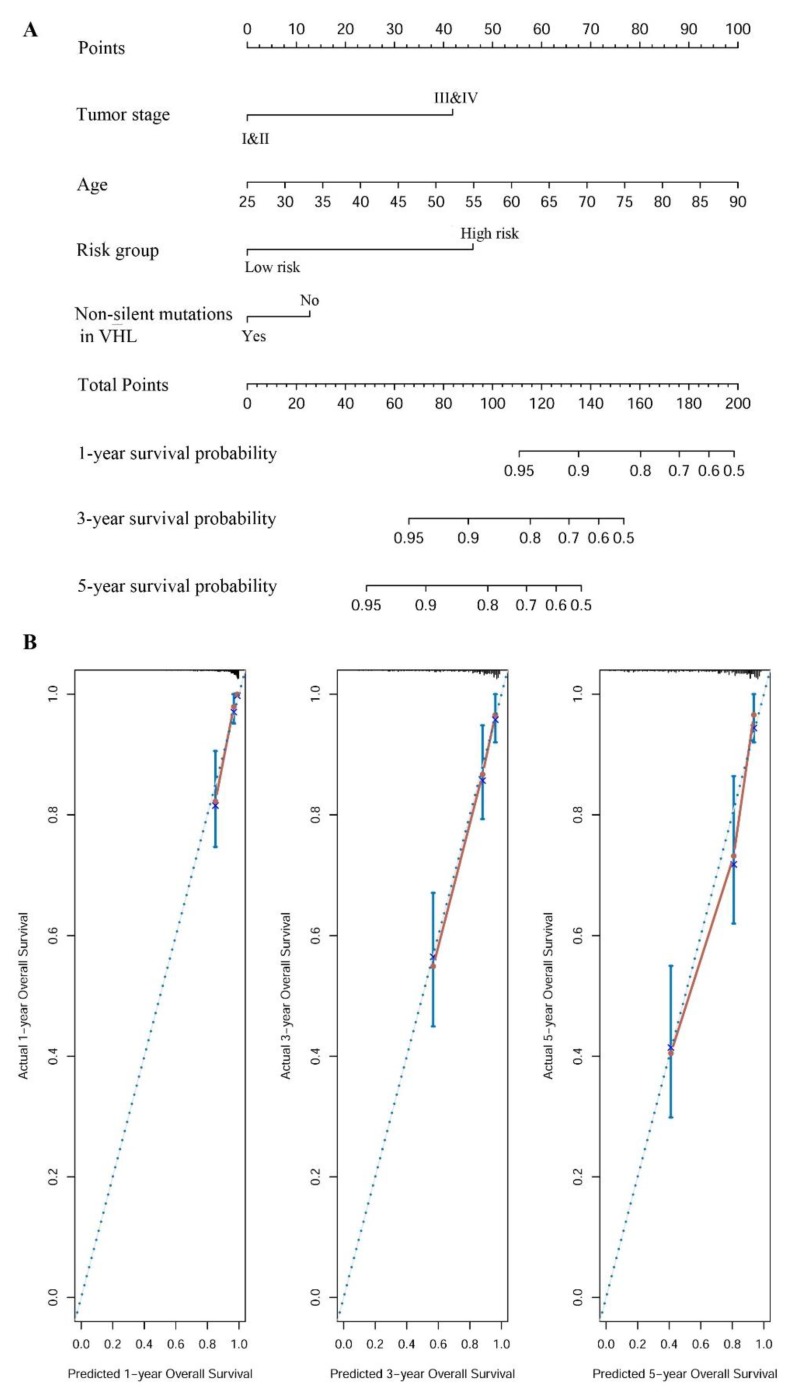
A nomogram for overall survival (OS) prediction in KIRC. (**A**) A nomogram for predicting OS in KIRC. This nomogram has four components: tumor stage, age, risk group, and non-silent mutation status of *VHL*. Each component corresponds to points according to the first axis “Points”. The total number of points of the four components of one patient lie on “Total Points” axis, which corresponds to the 1-, 3-, and 5-year survival probability that is plotted on the last three axes. (**B**) Calibration plots of the nomogram for predicting the OS rate at 1-, 3-, and 5-year survival, respectively.

Based on the best multivariate model, a prognostic nomogram was developed to predict the probabilities of 1-, 3-, and 5-year OS in KIRC using our defined risk group, tumor stage, age, and mutation status of *VHL* as variables (Figure 8A). Harrel’s concordance index (C-index) for OS prediction was 0.795, which suggests that our developed nomogram had high accuracy. The calibration curves for this nomogram were plotted (Figure 8B). They showed good agreement between the nomogram’s prediction and the actual observation for 1-, 3-, and 5-year OS rates. In addition, the predictive accuracy of the nomogram was compared with a system of other clinical characteristics, including sex, tumor stage, and age. The C-index of OS prediction was 0.53, 0.705, and 0.64 for sex, tumor stage, and age, respectively. These values were all lower than the C-index for our constructed nomogram (0.795). This indicates that our nomogram is a more accurate predictor of OS in KIRC than sex, tumor stage, or age.

## 3. Discussion

In this study, we developed a seven-MDEG signature to predict survival in KIRC patients. In brief, we first identified 863 DEGs with an altered DNA methylation status. Then, 189 MDEGs were found to be prognosis-related genes through univariate and multivariate Cox regression analyses. Seven MDEGs (*BID*, *CCNF*, *DLX4*, *FAM72D*, *PYCR1*, *RUNX1*, and *TRIP13*) of the 189 prognosis-related genes were selected to generate a risk score model by LASSO Cox regression. A survival analysis suggested that our seven-MDEG signature was an independent prognostic factor of KIRC. A best multivariate model that included our recorded risk group, tumor stage, age, and mutation status of *VHL* was obtained by a multivariate stepwise analysis. A prognostic nomogram was established for the best multivariate model, which showed its capability to predict survival for KIRC patients.

Differential expression genes were firstly identified using Student’s *t*-tests and fold- change (FC). To check whether different methods will make big difference to the results, we compared the DEGs found by Student’s *t*-test with the DEGs found by wilcoxon rank-sum test and DESeq2 [24] under the same cut-off levels, i.e., FDR<0.05 and $\left|\log_{2}(FC)\right|>1$. As it is shown in Figure S7, the DEGs detected by the three methods had high overlap rate, and only 37 DEGs (only accounting for 0.67%) found by Student’s *t*-test were not detected by wilcoxon rank-sum test or DESeq2, which suggests that using Student’s *t*-tests to detect DEGs is acceptable for the study. 

The 863 MDEGs were enriched in 29 KEGG pathways. Many of the enriched KEGG pathways play important roles in cancer development and progression. The PI3K-Akt signaling pathway is an intracellular signal transduction pathway of great importance in promoting metabolism, proliferation, cell survival, cell growth, and angiogenesis. The PI3K-Akt signaling pathway was reported to be a target for cancer treatment [25]. Guo et al. reported on the roles of the PI3K-Akt signaling pathway in renal cell carcinoma (RCC), and pointed out that the genes in the PI3K-Akt pathway are frequently mutation genes in KIRC [26], which indicates that the PI3K-Akt pathway may provide information that is helpful to the treatment of KIRC. Cell adhesion molecules (CAMs) mediate cell–cell and cell–extracellular matrix adhesion. Okegawa et al. reported on the roles of CAMs in cancer progression and discussed their potential for treating cancer, especially urogenital cancer [27]. *CADM4*, one of the CAMs, was identified as being a candidate tumor suppressor gene in RCC [28]. Changes in the structure of the extracellular matrix (ECM) mediate proliferation, differentiation, morphogenesis, and tumor progression [29]. The axon guidance pathway plays core roles in kidney development, and death can result from kidney abnormalities when it is abnormal [30]. The p53 pathway has been shown to be repressed in RCC by an unknown dominant mechanism [31]. Zhang et al. summarized the roles that Rap1 signaling plays in tumor cell invasion and metastasis [32]. A dysregulated carbon metabolism was found in KIRC [33]. Altered focal adhesion was discovered in a KIRC tumor [34]. The RAS signaling pathway was associated with colorectal cancer [35]. *HIF1-α* is related to the development of KIRC [36] and has prognostic value for KIRC [37]. The MAPK signaling pathway is implicated in cell proliferation and differentiation, and can suppress RCC when it is repressed [38].

Furthermore, we have also done the enrichment analysis for both 629 upregulated genes with hypo-methylated status and 234 downregulated genes with hyper-methylated status, respectively. The results were shown Figure S8, many enriched Gene Ontology (GO) and KEGG pathways were consistent with the enrichment analysis for all 863 MDEGs. It is worth mention that metabolic pathways were down-regulated which was also reported by Tun, et al. [39].

We identified seven MDEGs that were associated with the prognosis of KIRC from the TCGA discovery dataset. All seven genes have been associated with cancers. *BID* (BH3-interacting domain death agonist) is involved in extrinsic apoptotic signaling [40] and mediating the DNA damage response [41], and may be used as a therapeutic target [42]. *BID* is also a pro-apoptotic member of the B-cell lymphoma-2 (Bcl-2) group of proteins, which have a high expression level in RCC and may participate in the progression of cancer [43]. Moreover, *BID* was reported to be an independent prognostic gene in colon cancer [44]. Therefore, *BID* is crucial to KIRC and may be used as a prognosis biomarker for KIRC. Cyclin F, which is encoded by *CCNF*, can form the Skp1–Cul1–F-box E3 protein ubiquitin ligase complex. E3 ubiquitin ligases are implicated in ubiquitin–proteasome-mediated protein degradation. Low *CCNF* expression has been found in hepatocellular carcinoma (HCC) and is connected with a poor prognosis [45]. It was reported that *CCNF* controls tumorigenesis through IDH1-R132H [46]. High *CCNF* expression was associated with a poor outcome in melanoma patients [47]. An abnormal *DLX4* expression level has been reported in inflammatory breast cancer, leukemia, lung cancer, ovarian cancer, and prostate cancer [48,49,50,51,52]. Overexpression of *DLX4* leads to cancer migration, invasion, and metastasis by driving the expression of *TWIST* [53]. *FAM72D*, a family with sequence similarity 72 member D, can be considered to be a target gene in glioblastoma multiform (GBM) [54]. *FAM72D* was reported to be a new target for cancer therapies since its expression correlates with *UBE2C*, whose higher expression leads to a worse OS prognosis [55]. Pyrroline-5-carboxylate reductase 1 (*PYCR1*) can promote tumor cell growth in breast cancer [56]. *PYCR1* was reported to be overexpressed in prostate cancer [57]. Since *PYCR1* was found to be negatively regulated by miR-488, which is related to cell proliferation and tumorigenesis, it was reported to be a novel therapeutic target in lung cancer [58]. *PYCR1* may be a potential therapeutic target for treating prostate cancer and breast cancer [59,60]. Runt-related transcription factor 1 (*RUNX1*) has many roles in cancer. For example, *RUNX1* was found to be downregulated in gastric cancer [61] and hepatocellular carcinoma [62]. Genetic variation in *RUNX1* has been related to prostate cancer [63] and colorectal cancer [64]. A novel RUNX1–RUNX1T1 pathway was reported in KIRC, which may provide new ideas for treating KIRC [65]. The RUNX1/AKT pathway may be a new therapeutic target in kidney fibrosis [66]. Thyroid hormone Receptor Interactor 13 (*TRIP13*) was reported to be a prognostic biomarker for colorectal cancer [67] and prostate cancer [68]. Silencing *TRIP13* can activate TGF-β1/smad3 signaling, resulting in a repression of cell growth and metastasis of hepatocellular carcinoma (HCC) [69]. *TRIP13*-deficient tubular epithelial cells tend to progress towards apoptotic cell death following acute kidney injury [70].

During the univariant and multivariant survival analysis for MDEGs, no correction was carried out for multiple testing since we tried to keep all the possibly important genes, which was supported by the reference [71]. In the end, the LASSO Cox regression screened out the most informative genes (*BID*, *CCNF*, *DLX4*, *FAM72D*, *PYCR1*, *RUNX1*, and *TRIP13*). Actually, we checked the adjust *p*-values of the seven genes. In the univariant analysis, the adjust *p*-values for *BID*, *CCNF*, *DLX4*, *FAM72D*, *PYCR1*, *RUNX1*, and *TRIP13* were 6.80 × 10^−3^, 2.00 × 10^−3^, 0, 2.57 × 10^−2^, 7.00 × 10^−4^, 1.50 × 10^−3^ and 2.80 × 10^−3^, respectively. In the multivariate analysis, the adjust *p*-values for the seven genes were 6.27 × 10^−2^, 1.29 × 10^−2^, 7.53 × 10^−3^, 3.84 × 10^−2^, 5.92 × 10^−3^, 7.53 × 10^−3^, and 3.68 × 10^−2^, respectively. Almost all the *p*-values are less than 0.05, except one (i.e. 6.27E-2, *BID* in multivariate analysis). However, as we discussed above, *BID* is an important gene for KIRC.

In our LASSO Cox regression, seven MDEGs were selected in the end which met the one in ten rule [72] since we have 72 patients who died. And through multivariate stepwise analysis, a best multivariate model with four predictors was obtained, which also satisfied the rule that a minimum of 10 outcome events per predictor variable (EPV) since we had 72 patients who died. In the multivariate Cox regression analysis for each MDEG, nine predictors were included in the model to investigate the influences of the eight confounding factors on the MDEG. According to the study of Vittinghoff E. [73], one in 10 rule can be relaxed. They suggested that five to nine events per predictor can be enough. Our study met this requirement.

Our univariate Cox regression analysis showed that *VHL* mutation was not related to survival in KIRC patients, which is consistent with the research done by Kim et al. [8]. We also found that *BAP1* mutation has some prognostic value in KIRC patients, which is consistent with the report done by Wang et al. [9]. Although the mutation statue of *VHL* was not shown in the univariate Cox regression analysis to have prognostic value, it was contained in the best multivariate model together with our recorded risk group, suggesting that it has a potential role in prognosis for KIRC patients.

Since we cannot find the KIRC data with both RNA-seq and clinical data from the public database, we used KIRP (kidney renal papillary cell carcinoma) data from TCGA as an external validation dataset. KIRP and KIRC share many characteristics since both of them are subtypes of kidney cancer and originate from the same tissue. The results showed that our risk score model achieved a good result in KIRP data (Figure S9). Significant survival differences between the high-risk subgroup and the low-risk subgroup were observed in the KIRP dataset (Figure S9A; the *p*-value of the log-rank is 5.00 × 10^−4^). The receiver operating characteristic (ROC) analysis showed that the risk score based on the seven MDEGs performed well in death prediction (Figure S9B). The area under the curve (AUC) at 1, 3, and 5 years was 0.871, 0.796, and 0.719, respectively.

The main limitations of this paper are: (1) the seven-gene signature was generated from the TCGA dataset, in which most patients are Caucasian; and (2) this signature was only validated in the TCGA cohort. Therefore, this signature needs to be further investigated in multiple datasets with different populations.

## 4. Materials and Methods

### 4.1. Datasets and Networks

The multiplatform genomics datasets including gene expression, DNA methylation, and somatic mutation information were downloaded from the Cancer Genome Atlas (TCGA, http://cancergenome.nih.gov/) for KIRC. The clinical information was obtained through the TCGA Data Commons (https://gdc.cancer.gov/).

### 4.2. Identification of Differentially Expressed Genes (DEGs) with an Altered DNA Methylation Status in KIRC

Gene expression data and DNA methylation data were employed to identify DEGs with an altered methylation status. Firstly, Student’s *t*-test and fold-change (FC) were used to determine DEGs in KIRC. Next, the DNA methylation status of genes was compared between KIRC samples and matched normal samples by using the Chip Analysis of Methylation Pipeline (ChAMP) [74,75] available in the R Bioconductor package. Then, both downregulated genes with a hypermethylated status and upregulated genes with a hypomethylated status were identified as candidate genes for prognosis.

### 4.3. Functional and Pathway Enrichment Analyses

The GO functional enrichment analysis and the KEGG pathway enrichment analysis were performed using the DAVID platform [76] for the downregulated genes with a hypermethylated status and upregulated genes with a hypomethylated status.

### 4.4. Establishment of the MDEG Signature for Prognosis of KIRC

For each MDEG, all patients were divided into high- and low-expression subgroups by using the median expression of the gene as a cut-off. The association between MDEG expression and patients’ OS was assessed by a Kaplan–Meier (KM) survival curve and a log-rank test between subgroups of high- and low-expression patients for each MDEG. MDEGs that show obvious differences between two groups can be used as potential prognosis biomarkers for cancer. Furthermore, multivariate Cox models (function “coxph”, R package “survival”) were further used to investigate the influences of the confounding factors on MDEGs. The confounding factors included sex information, pathologic stages of a tumor, age at initial pathologic diagnosis, and the mutation status of genes in KIRC. MDEGs with a *p*-value of less than 0.05 in the log-rank test for both the univariate analysis and the multivariate analysis were further screened and confirmed by LASSO Cox regression analysis. Then, independent MDEG biomarkers were integrated into an MDEG signature by calculating a risk score with the following formula:

(2) $$RS=\sum_{i=1}^{n}Coefficient\;of\;Gene\;(i)*Expression\;of\;Gene(i)$$

where “*RS*”, which is short for “risk score”, is a MDEG signature risk score for each KIRC patient; *Coefficient of Gene (i)* is the regression coefficient of Gene *(i)* in the best LASSO Cox regression model; and *Expression of Gene (i)* is the expression value of Gene *(i)* for the patient. Based on this formula, a MDEG signature risk score can be obtained for each patient. Then, the median risk score was used as the cutoff to divide patients into a high-risk subgroup and a low-risk subgroup. A KM survival curve analysis and a log-rank test was then performed to compare survival between the high- and low-risk subgroups.

### 4.5. Statistical Analysis

To evaluate the prognostic performance of the risk score system, univariate and multivariate models were computed using Cox proportional-hazards regression. The univariate analysis was used to independently assess the prognostic performance of each variable (Table 1). Moreover, a multivariate model including risk group (high-risk group and low-risk group) and all of the other variables (Table 1) was developed. Then, to select the most informative variables, a backward–forward step procedure was carried out in the R package “stats”. Furthermore, a nomogram was constructed for the best multivariate model using the R package “rms”. The performance of the nomogram was assessed with Harrel’s concordance index (C-index), which is a useful evaluation measure that is similar to calculating the area under the receiver operating characteristic (ROC) curve. C-indices range from 0.5 to 1.0, where 0.5 denotes a bad performance and 1 denotes a good performance [77]. Calibration curves were drawn to check for consistency between the predicted survival and the observed survival.

## 5. Conclusions

In conclusion, we built a seven-MDEG signature for predicting survival in KIRC patients by making full use of multi-platform datasets including DNA methylation data, gene expression profiles, somatic mutation data, and clinical information. Our results showed that this signature is an independent prognostic factor in KIRC patients and can more accurately predict OS in KIRC patients than a tumor stage system. Therefore, our constructed signature has potential application in realizing future personalized medicine for KIRC patients.

## Figures and Tables

**Table 1:** Patient and tumor characteristics.

Characteristic	TCGA Discovery Dataset (*n* = 330)		TCGA Validation Dataset (*n* = 196)	
	No.	%	No.	%
Status				
Alive	258	78.2	109	55.6
Dead	72	21.8	87	44.4
Sex				
Male	214	64.8	127	64.8
Female	116	35.2	69	35.2
Tumor stage				
Stages I and II	222	67.3	98	50.0
Stages III and IV	108	32.7	98	50.0
Age, years				
Median	60	—	62	—
Range	26–90	—	34–85	—
Non-silent mutations in *VHL* (Von Hippel–Lindau Tumor Suppressor)				
Yes	150	45.5	—	—
No	180	54.5	—	—
Non-silent mutations in *PBRM1* (Polybromo 1)				
Yes	128	38.8	—	—
No	202	61.2	—	—
Non-silent mutations in *TTN* (Titin)				
Yes	58	17.6	—	—
No	272	82.4	—	—
Non-silent mutations in *SETD2* (SET Domain Containing 2)				
Yes	39	11.8	—	—
No	291	88.2	—	—
Non-silent mutations in *BAP1* (BRCA1 Associated Protein 1)				
Yes	29	8.8	—	—
No	301	91.2	—	—

**Table 2:** Seven MDEGs associated with KIRC prognosis.

No.	Genes	Description	Univariate Analysis	Multivariate Analysis
			Modality *p* Value	Modality *p* Value
1	*BID*	BH3 Interacting Domain Death Agonist	6.50 × 10^−4^	0.025
2	*CCNF*	Cyclin F	8.00 × 10^−5^	0.001
3	*DLX4*	Distal-Less Homeobox 4	0	<0.001
4	*FAM72D*	Family with Sequence Similarity 72 Member D	4.74 × 10^−3^	0.011
5	*PYCR1*	Pyrroline-5-Carboxylate Reductase 1	1.00 × 10^−5^	<0.001
6	*RUNX1*	Runt Related Transcription Factor 1	4.00 × 10^−5^	<0.001
7	*TRIP13*	Thyroid Hormone Receptor Interactor 13	1.40 × 10^−4^	0.009

**Table 3:** Univariate and multivariate Cox analyses including the risk group and other molecular annotations.

Variables	Univariate Analysis					Best Multivariate Model			
	Value	HR	95%C.I.	Modality	Model	HR	95%C.I.	modality	model
				*p* Value	*p* Value			*p* Value	*p* Value
				(Wald)	(Log-Rank)			(Wald)	(Log-Rank)
Tumor stage (ref = I and II)	III and IV	4.69	2.84–7.75	1.48 × 10^−9^	3.00 × 10^−11^	3.47	2.06–5.84	2.80 × 10^−6^	<2.00 × 10^−16^
Age	—	1.04	1.02–1.06	2.25 × 10^−5^	2.00 × 10^−5^	1.04	1.02–1.07	3.62 × 10^−5^	
Risk group (Ref = low risk)	High risk	5.15	2.81–9.45	1.17 × 10^−7^	4.00 × 10^−9^	3.92	2.08–7.38	2.25 × 10^−5^	
Non-silent mutations in *VHL* (ref = No)	Yes	0.77	0.48–1.23	0.28	0.3	0.68	0.43–1.10	0.11	
Non-silent mutations in *PBRM1* (ref = No)	Yes	0.83	0.51–1.34	0.44	0.4				
Non-silent mutations in *TTN* (ref = No)	Yes	1.24	0.70–2.21	0.46	0.5				
Non-silent mutations in *SETD2* (ref = No)	Yes	1.44	0.77–2.69	0.25	0.2				
Non-silent mutations in *BAP1* (ref = No)	Yes	2.05	1.15–3.65	0.01	0.01				
Sex (ref = Female)	Male	0.75	0.47–1.20	0.22	0.2				

HR, Cox hazard ratio; 95% C.I., 95% confidence interval of the HR; Value, modality of the annotation associated with the HR; ref, reference.

## Supplementary Materials

Supplementary materials can be found at https://www.mdpi.com/1422-0067/20/22/5720/s1.

## References

[1] Shuch B., Amin A., Armstrong A.J., Eble J.N., Ficarra V., Lopez-Beltran A., Martignoni G., Rini B.I., Kutikov A. (2015). Understanding pathologic variants of renal cell carcinoma: Distilling therapeutic opportunities from biologic complexity. Eur. Urol..

[2] Gray E.R., Harris G.T. (2019). Renal Cell Carcinoma: Diagnosis and Management. Am. Fam. Phys..

[3] Shen C., Kaelin W.G. (2013). The VHL/HIF axis in clear cell renal carcinoma. Semin. Cancer Biol..

[4] Nyhan M.J., O’Sullivan G.C., McKenna S.L. (2008). Role of the VHL (von Hippel-Lindau) gene in renal cancer: A multifunctional tumour suppressor. Biochem. Soc. Trans..

[5] Atkins M.B., Tannir N.M. (2018). Current and emerging therapies for first-line treatment of metastatic clear cell renal cell carcinoma. Cancer Treat. Rev..

[6] Alonso-Gordoa T., Garcia-Bermejo M.L., Grande E., Garrido P., Carrato A., Molina-Cerrillo J. (2019). Targeting tyrosine kinases in renal cell carcinoma: ‘New Bullets against Old Guys’. Int. J. Mol. Sci..

[7] Bihr S., Ohashi R., Moore A.L., Ruschoff J.H., Beisel C., Hermanns T., Mischo A., Corro C., Beyer J., Beerenwinkel N. (2019). Expression and mutation patterns of PBRM1, BAP1 and SETD2 mirror specific evolutionary subtypes in clear cell renal cell carcinoma. Neoplasia.

[8] Kim B.J., Kim J.H., Kim H.S., Zang D.Y. (2017). Prognostic and predictive value of *VHL* gene alteration in renal cell carcinoma: A meta-analysis and review. Oncotarget.

[9] Wang X.Y., Wang Z., Huang J.B., Ren X.D., Ye D., Zhu W.W., Qin L.X. (2017). Tissue-specific significance of BAP1 gene mutation in prognostic prediction and molecular taxonomy among different types of cancer. Tumour Biol..

[10] Liu L., Guo R.B., Zhang X., Liang Y.R., Kong F., Wang J., Xu Z.H. (2017). Loss of SETD2, but not H3K36me3, correlates with aggressive clinicopathological features of clear cell renal cell carcinoma patients. Biosci. Trends.

[11] Wang Z., Peng S., Guo L., Xie H., Wang A., Shang Z., Niu Y. (2018). Prognostic and clinicopathological value of PBRM1 expression in renal cell carcinoma. Clin. Chim. Acta.

[12] Frank I., Blute M.L., Cheville J.C., Lohse C.M., Weaver A.L., Zincke H. (2002). An outcome prediction model for patients with clear cell renal cell carcinoma treated with radical nephrectomy based on tumor stage, size, grade and necrosis: The SSIGN score. J. Urol..

[13] Martinez-Salamanca J.I., Huang W.C., Millan I., Bertini R., Bianco F.J., Carballido J.A., Ciancio G., Hernandez C., Herranz F., Haferkamp A. (2011). International renal cell carcinoma-venous thrombus, prognostic impact of the 2009 UICC/AJCC TNM staging system for renal cell carcinoma with venous extension. Eur. Urol..

[14] Li P., Liu J., Li J., Liu P. (2019). DNA methylation of CRB3 is a prognostic biomarker in clear cell renal cell carcinoma. Mol. Biol. Rep..

[15] Ricketts C.J., Hill V.K., Linehan W.M. (2014). Tumor-Specific hypermethylation of epigenetic biomarkers, including SFRP1, predicts for poorer survival in patients from the TCGA Kidney Renal Clear Cell Carcinoma (KIRC) project. PLoS ONE.

[16] Kim Y.J., Jang W., Piao X.M., Yoon H.Y., Byun Y.J., Kim J.S., Kim S.M., Lee S.K., Seo S.P., Kang H.W. (2019). ZNF492 and GPR149 methylation patterns as prognostic markers for clear cell renal cell carcinoma: Arraybased DNA methylation profiling. Oncol. Rep..

[17] Wang Y., Ruan Z., Yu S., Tian T., Liang X., Jing L., Li W., Wang X., Xiang L., Claret F.X. (2019). A four-methylated mRNA signature-based risk score system predicts survival in patients with hepatocellular carcinoma. Aging.

[18] Chen H.M., Kong Y., Yao Q., Zhang X., Fu Y.N., Li J., Liu C., Wang Z. (2019). Three hypomethylated genes were associated with poor overall survival in pancreatic cancer patients. Aging.

[19] Gao Z., Zhang D., Duan Y., Yan L., Fan Y.D., Fang Z.Q., Liu Z.X. (2019). A five-gene signature predicts overall survival of patients with papillary renal cell carcinoma. PLoS ONE.

[20] Wu J., Jin S., Gu W., Wan F., Zhang H., Shi G., Qu Y., Ye D. (2019). Construction and validation of a 9-gene signature for predicting prognosis in stage III clear cell renal cell carcinoma. Front. Oncol..

[21] Huang Z., Zhan X., Xiang S., Johnson T.S., Helm B., Yu C.Y., Zhang J., Salama P., Rizkalla M., Han Z. (2019). SALMON: Survival analysis learning with multi-omics neural networks on breast cancer. Front. Genet..

[22] Mishra N.K., Southekal S., Guda C. (2019). Survival analysis of multi-omics data identifies potential prognostic markers of pancreatic ductal adenocarcinoma. Front. Genet..

[23] Benjamini Y., Hochberg Y. (1995). Controlling the false discovery rate: A practical and powerful approach to multiple testing. J. Royal Stat. Soc. Ser. B.

[24] Love M.I., Huber W., Anders S. (2014). Moderated estimation of fold change and dispersion for RNA-seq data with DESeq2. Genome Biol..

[25] Martini M., de Santis M.C., Braccini L., Gulluni F., Hirsch E. (2014). PI3K/AKT signaling pathway and cancer: An updated review. Ann. Med..

[26] Guo H., German P., Bai S., Barnes S., Guo W., Qi X., Lou H., Liang J., Jonasch E., Mills G.B. (2015). The PI3K/AKT pathway and renal cell carcinoma. J. Genet. Genom..

[27] Okegawa T., Pong R.C., Li Y., Hsieh J.T. (2004). The role of cell adhesion molecule in cancer progression and its application in cancer therapy. Acta Biochim. Pol..

[28] Nagata M., Sakurai-Yageta M., Yamada D., Goto A., Ito A., Fukuhara H., Kume H., Morikawa T., Fukayama M., Homma Y. (2012). Aberrations of a cell adhesion molecule CADM4 in renal clear cell carcinoma. Int. J. Cancer.

[29] Pupa S.M., Menard S., Forti S., Tagliabue E. (2002). New insights into the role of extracellular matrix during tumor onset and progression. J. Cell. Physiol..

[30] Grieshammer U., Le M., Plump A.S., Wang F., Tessier-Lavigne M., Martin G.R. (2004). SLIT2-mediated ROBO2 signaling restricts kidney induction to a single site. Dev. Cell.

[31] Gurova K.V., Hill J.E., Razorenova O.V., Chumakov P.M., Gudkov A.V. (2004). P53 pathway in renal cell carcinoma is repressed by a dominant mechanism. Cancer Res..

[32] Zhang Y.L., Wang R.C., Cheng K., Ring B.Z., Su L. (2017). Roles of Rap1 signaling in tumor cell migration and invasion. Cancer Biol. Med..

[33] Hakimi A.A., Reznik E., Lee C.H., Creighton C.J., Brannon A.R., Luna A., Aksoy B.A., Liu E.M., Shen R., Lee W. (2016). An integrated metabolic atlas of clear cell renal cell carcinoma. Cancer Cell.

[34] Maziveyi M., Alahari S.K. (2017). Cell matrix adhesions in cancer: The proteins that form the glue. Oncotarget.

[35] Zenonos K., Kyprianou K. (2013). RAS signaling pathways, mutations and their role in colorectal cancer. World J. Gastrointest. Oncol..

[36] Gudas L.J., Fu L., Minton D.R., Mongan N.P., Nanus D.M. (2014). The role of HIF1α in renal cell carcinoma tumorigenesis. J. Mol. Med..

[37] Salinas-Sanchez A.S., Serrano-Oviedo L., Nam-Cha S.Y., Roche-Losada O., Sanchez-Prieto R., Gimenez-Bachs J.M. (2017). Prognostic value of the VHL, HIF-1 alpha, and VEGF signaling pathway and associated MAPK (ERK1/2 and ERK5) pathways in clear-cell renal cell carcinoma: A long-term study. Clin. Genitourin. Cancer.

[38] Huang D., Ding Y., Luo W.M., Bender S., Qian C.N., Kort E., Zhang Z.F., VandenBeldt K., Duesbery N.S., Resau J.H. (2008). Inhibition of MAPK kinase signaling pathways suppressed renal cell carcinoma growth and angiogenesis in vivo. Cancer Res..

[39] Tun H.W., Marlow L.A., von Roemeling C.A., Cooper S.J., Kreinest P., Wu K., Luxon B.A., Sinha M., Anastasiadis P.Z., Copland J.A. (2010). Pathway signature and cellular differentiation in clear cell renal cell carcinoma. PLoS ONE.

[40] Kantari C., Walczak H. (2011). Caspase-8 and Bid: Caught in the act between death receptors and mitochondria. Biochimica Biophysica Acta Mol. Cell Res..

[41] Liu Y., Bertram C.C., Shi Q., Zinkel S.S. (2011). Proapoptotic Bid mediates the Atr-directed DNA damage response to replicative stress. Cell Death Differ..

[42] Orzechowska E.J., Girstun A., Staron K., Trzcinska-Danielewicz J. (2015). Synergy of BID with doxorubicin in the killing of cancer cells. Oncol. Rep..

[43] Gobe G., Rubin M., Williams G., Sawczuk I., Buttyan R. (2002). Apoptosis and expression of Bcl-2, Bcl-XL, and Bax in renal cell carcinomas. Cancer Investig..

[44] Sinicrope F.A., Rego R.L., Foster N.R., Thibodeau S.N., Alberts S.R., Windschitl H.E., Sargent D.J. (2008). Proapoptotic Bad and Bid protein expression predict survival in stages II and III colon cancers. Clin. Cancer Res..

[45] Fu J., Qiu H., Cai M., Pan Y., Cao Y., Liu L., Yun J., Zhang C.Z. (2013). Low cyclin F expression in hepatocellular carcinoma associates with poor differentiation and unfavorable prognosis. Cancer Sci..

[46] Deshmukh R.S., Das S. (2017). Cyclin F controls glioma progression by regulation of IDH1-R132H expression. Ann. Oncol..

[47] Gagat M., Krajewski A., Grzanka D., Grzanka A. (2018). Potential role of cyclin F mRNA expression in the survival of skin melanoma patients: Comprehensive analysis of the pathways altered due to cyclin F upregulation. Oncol. Rep..

[48] Jeong J., Naab T.J., Fernandez A.I., Ongkeko M.S., Makambi K.H., Blancato J.K. (2018). Homeoprotein DLX4 expression is increased in inflammatory breast cancer cases from an urban African-American population. Oncotarget.

[49] Haga S.B., Fu S., Karp J.E., Ross D.D., Williams D.M., Hankins W.D., Behm F., Ruscetti F.W., Chang M., Smith B.D. (2000). *BP1*, a new homeobox gene, is frequently expressed in acute leukemias. Leukemia.

[50] Schwartz A.M., Man Y.G., Rezaei M.K., Simmens S.J., Berg P.E. (2009). BP1, a homeoprotein, is significantly expressed in prostate adenocarcinoma and is concordant with prostatic intraepithelial neoplasia. Mod. Pathol..

[51] Hara F., Samuel S., Liu J., Rosen D., Langley R.R., Naora H. (2007). A homeobox gene related to Drosophila distal-less promotes ovarian tumorigenicity by inducing expression of vascular endothelial growth factor and fibroblast growth factor-2. Am. J. Pathol..

[52] Yu M., Wan Y., Zou Q. (2008). Prognostic significance of BP1 mRNA expression level in patients with non-small cell lung cancer. Clin. Biochem..

[53] Zhang L., Yang M., Gan L., He T., Xiao X., Stewart M.D., Liu X., Yang L., Zhang T., Zhao Y. (2012). DLX4 upregulates TWIST and enhances tumor migration, invasion and metastasis. Int. J. Biol. Sci..

[54] Rahane C.S., Kutzner A., Heese K. (2019). A cancer tissue-specific FAM72 expression profile defines a novel glioblastoma multiform (GBM) gene-mutation signature. J. Neurooncol..

[55] Dastsooz H., Cereda M., Donna D., Oliviero S. (2019). A comprehensive bioinformatics analysis of UBE2C in cancers. Int. J. Mol. Sci..

[56] Possemato R., Marks K.M., Shaul Y.D., Pacold M.E., Kim D., Birsoy K., Sethumadhavan S., Woo H.K., Jang H.G., Jha A.K. (2011). Functional genomics reveal that the serine synthesis pathway is essential in breast cancer. Nature.

[57] Ernst T., Hergenhahn M., Kenzelmann M., Cohen C.D., Bonrouhi M., Weninger A., Klaren R., Grone E.F., Wiesel M., Gudemann C. (2002). Decrease and gain of gene expression are equally discriminatory markers for prostate carcinoma: A gene expression analysis on total and microdissected prostate tissue. Am. J. Pathol..

[58] Wang D., Wang L., Zhang Y., Yan Z., Liu L., Chen G. (2019). PYCR1 promotes the progression of non-small-cell lung cancer under the negative regulation of miR-488. Biomed. Pharmacother..

[59] Zeng T., Zhu L., Liao M., Zhuo W., Yang S., Wu W., Wang D. (2017). Knockdown of PYCR1 inhibits cell proliferation and colony formation via cell cycle arrest and apoptosis in prostate cancer. Med. Oncol..

[60] Ding J., Kuo M.L., Su L., Xue L., Luh F., Zhang H., Wang J., Lin T.G., Zhang K., Chu P. (2017). Human mitochondrial pyrroline-5-carboxylate reductase 1 promotes invasiveness and impacts survival in breast cancers. Carcinogenesis.

[61] Sakakura C., Hagiwara A., Mivagawa K., Nakashima S., Yoshikawa T., Kin S., Nakase Y., Ito K., Yamagishi H., Yazumi S. (2005). Frequent downregulation of the runt domain transcription factors RUNX1, RUNX3 and their cofactor CBFB in gastric cancer. Int. J. Cancer.

[62] Miyagawa K., Sakakura C., Nakashima S., Yoshikawa T., Kin S., Nakase Y., Ito K., Yamagishi H., Ida H., Yazumi S. (2006). Down-regulation of RUNX1, RUNX3 and CBFbeta in hepatocellular carcinomas in an early stage of hepatocarcinogenesis. Anticancer Res..

[63] Huang S.P., Lan Y.H., Lu T.L., Pao J.B., Chang T.Y., Lee H.Z., Yang W.H., Hsieh C.J., Chen L.M., Huang L.C. (2011). Clinical significance of runt-related transcription factor 1 polymorphism in prostate cancer. BJU Int..

[64] Slattery M.L., Lundgreen A., Herrick J.S., Caan B.J., Potter J.D., Wolff R.K. (2011). Associations between genetic variation in RUNX1, RUNX2, RUNX3, MAPK1 and eIF4E and risk of colon and rectal cancer: Additional support for a TGF-beta-signaling pathway. Carcinogenesis.

[65] Xiong Z., Yu H., Ding Y., Feng C., Wei H., Tao S., Huang D., Zheng S.L., Sun J., Xu J. (2014). RNA sequencing reveals upregulation of RUNX1-RUNX1T1 gene signatures in clear cell renal cell carcinoma. Biomed. Res. Int..

[66] Galichon P. (2018). Epithelial signaling through the RUNX1/AKT Pathway: A new therapeutic target in kidney fibrosis. Ebiomedicine.

[67] Sheng N.Q., Yan L., Wu K., You W.Q., Gong J.F., Hu L.D., Tan G.W., Chen H.Q., Wang Z.G. (2018). TRIP13 promotes tumor growth and is associated with poor prognosis in colorectal cancer. Cell Death Dis..

[68] Dong L.M., Ding H.L., Li Y.P., Xue D.W., Li Z., Liu Y.L., Zhang T., Zhou J., Wang P. (2019). TRIP13 is a predictor for poor prognosis and regulates cell proliferation, migration and invasion in prostate cancer. Int. J. Biol. Macromol..

[69] Yao J.N., Zhang X.X., Li J.H., Zhao D.Y., Gao B., Zhou H.N., Gao S.L., Zhang L.F. (2018). Silencing TRIP13 inhibits cell growth and metastasis of hepatocellular carcinoma by activating of TGF-beta 1/smad3. Cancer Cell Int..

[70] Pressly J.D., Hama T., Brien S.O., Regner K.R., Park F. (2017). TRIP13-deficient tubular epithelial cells are susceptible to apoptosis following acute kidney injury. Sci. Rep..

[71] Rothman K.J. (1990). No adjustments are needed for multiple comparisons. Epidemiology.

[72] Peduzzi P., Concato J., Feinstein A.R., Holford T.R. (1995). Importance of events per independent variable in proportional hazards regression analysis. II. Accuracy and precision of regression estimates. J. Clin. Epidemiol..

[73] Vittinghoff E., McCulloch C.E. (2007). Relaxing the rule of ten events per variable in logistic and cox regression. Am. J. Epidemiol..

[74] Tian Y., Morris T.J., Webster A.P., Yang Z., Beck S., Feber A., Teschendorff A.E. (2017). ChAMP: Updated methylation analysis pipeline for Illumina BEADCHIPS. Bioinformatics.

[75] Morris T.J., Butcher L.M., Feber A., Teschendorff A.E., Chakravarthy A.R., Wojdacz T.K., Beck S. (2014). ChAMP: 450k chip analysis methylation pipeline. Bioinformatics.

[76] Huang Da W., Sherman B.T., Lempicki R.A. (2009). Systematic and integrative analysis of large gene lists using DAVID bioinformatics resources. Nat. Protoc..

[77] Valentini V., van Stiphout R.G., Lammering G., Gambacorta M.A., Barba M.C., Bebenek M., Bonnetain F., Bosset J.F., Bujko K., Cionini L. (2011). Nomograms for predicting local recurrence, distant metastases, and overall survival for patients with locally advanced rectal cancer on the basis of European randomized clinical trials. J. Clin. Oncol..

