# Supplementary material for: A Gene Signature of Survival Prediction for Kidney Renal Cell Carcinoma by Multi-Omic Data Analysis
Source: Int J Mol Sci. 2019 Nov 14;20(22):5720. doi: 10.3390/ijms20225720 (PMC6888680; doi:10.3390/ijms20225720)
Supplement: Supplementary file 1 [file ijms-20-05720-s001.pdf]

Supplementary Figures

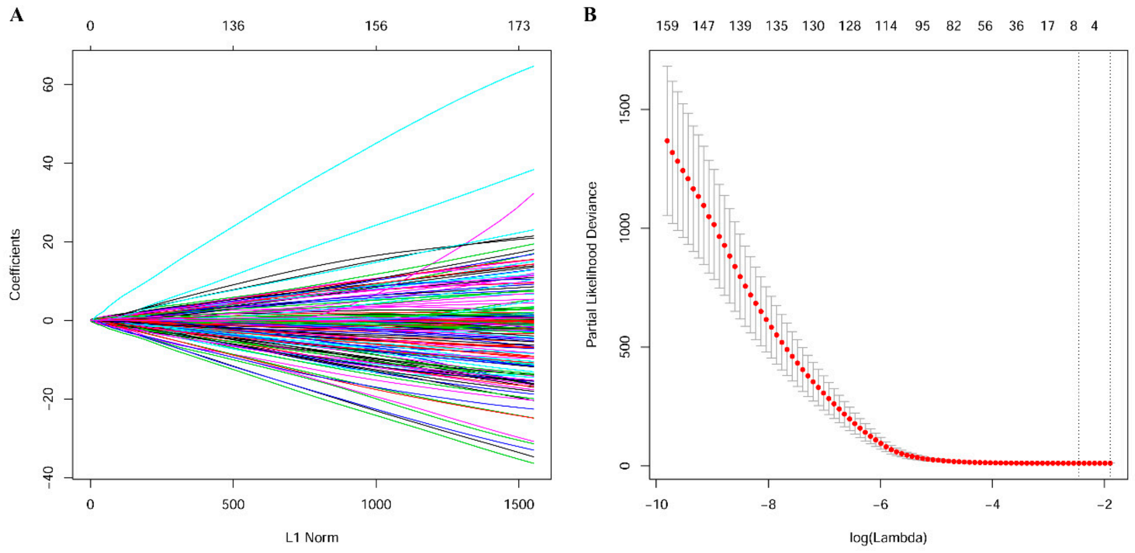

Figure S1. LASSO regression results. (A) Plot of LASSO coefficient profiles. (B) Plot of partial likelihood deviance for the 189 MDEGs in TCGA discovery cohort.

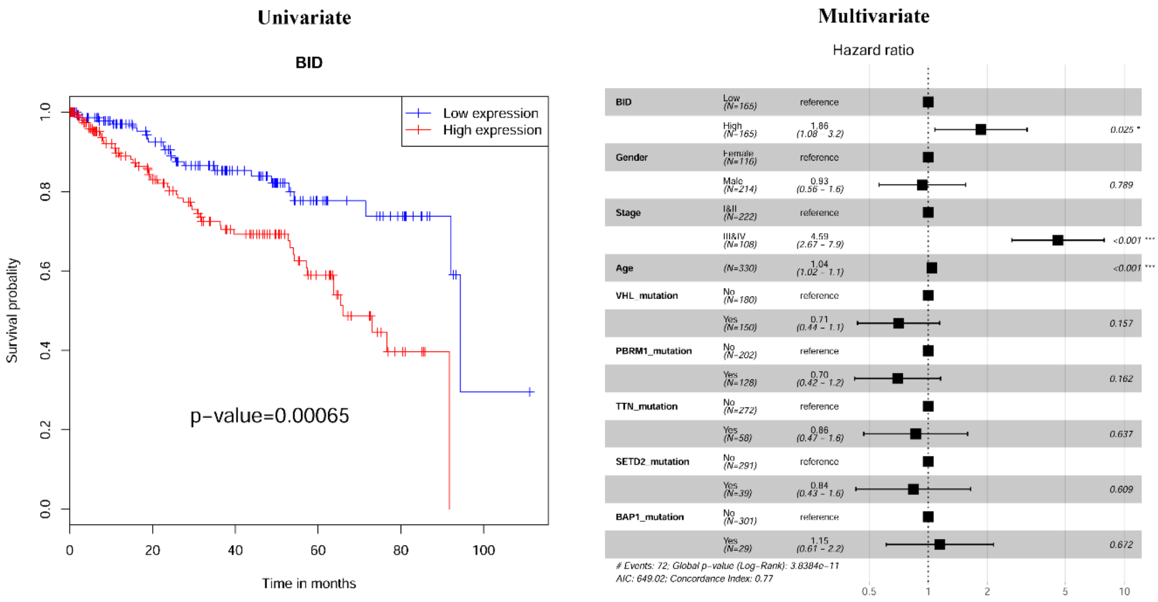

Figure S2. The expression of *BID* associated with survival outcomes in KIRC. Kaplan-Meier curves showed that patients with high expression of *BID* had worse

outcome compared with those with low expression of *BID*. Multivariate Cox regression analysis showed that *BID* was an independent prognostic factor.

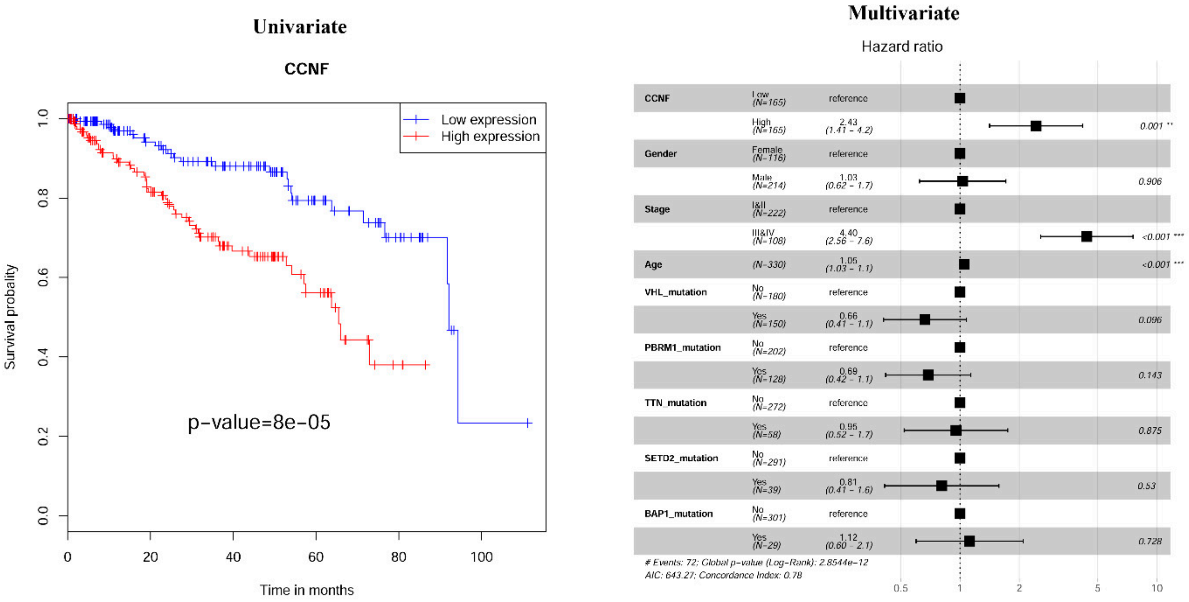

Figure S3. The expression of *CCNF* associated with survival outcomes in KIRC. Kaplan-Meier curves showed that patients with high expression of *CCNF* had worse outcome compared with those with low expression of *CCNF*. Multivariate Cox regression analysis showed that *CCNF* was an independent prognostic factor.

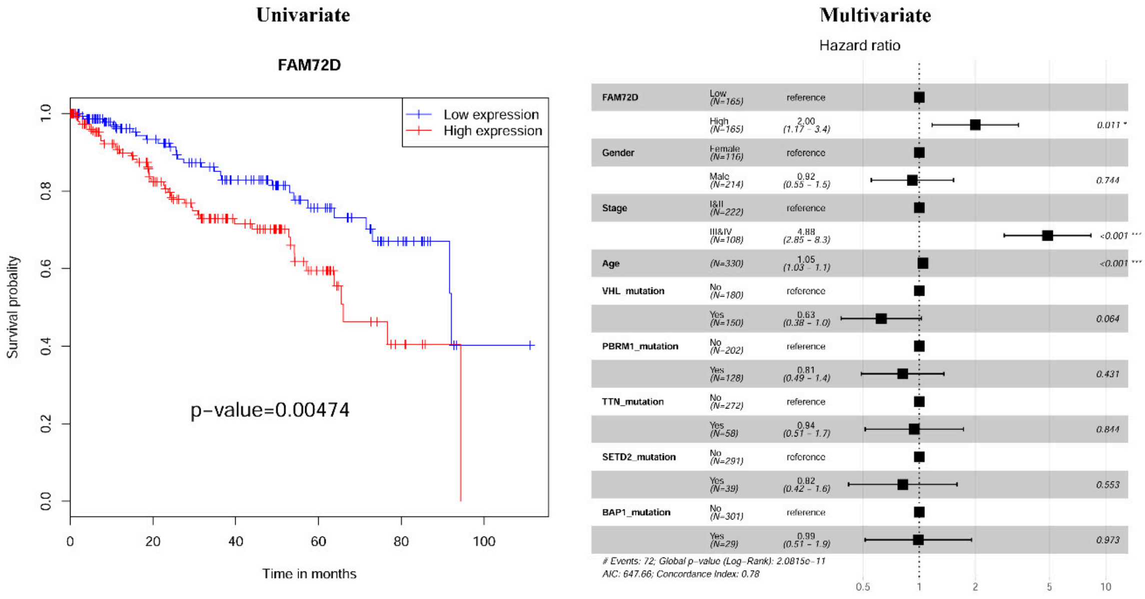

Figure S4. The expression of *FAM72D* associated with survival outcomes in KIRC. Kaplan-Meier curves showed that patients with high expression of *FAM72D* had worse outcome compared with those with low expression of *FAM72D*. Multivariate Cox regression analysis showed that *FAM72D* was an independent prognostic factor.

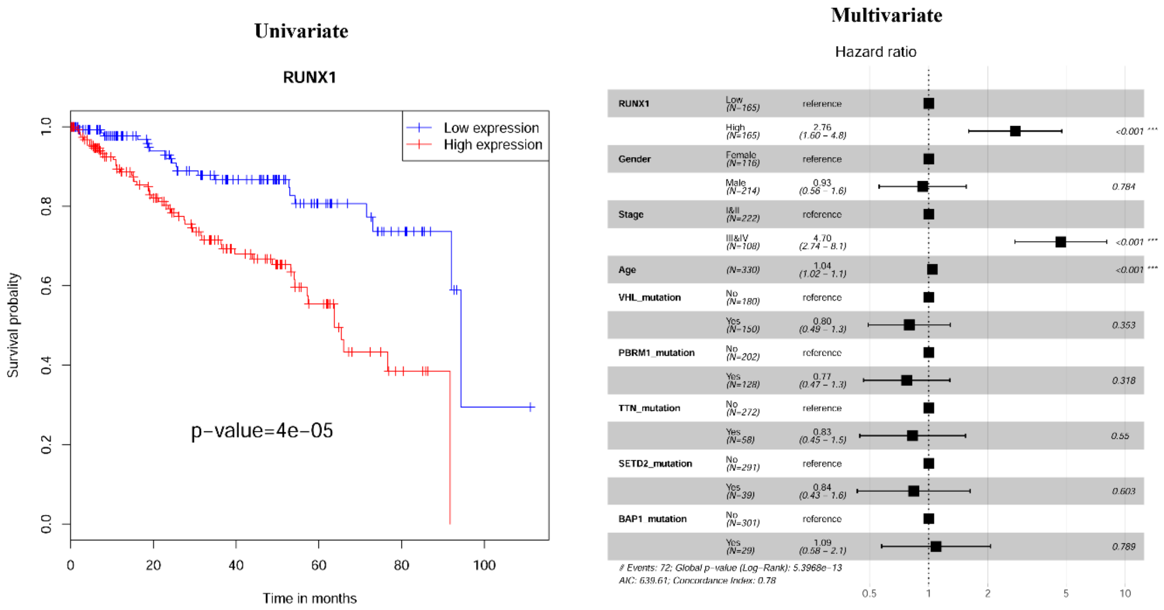

Figure S5. The expression of *RUNX1* associated with survival outcomes in KIRC. Kaplan-Meier curves showed that patients with high expression of *RUNX1* had worse outcome compared with those with low expression of *RUNX1*. Multivariate Cox regression analysis showed that *RUNX1* was an independent prognostic factor.

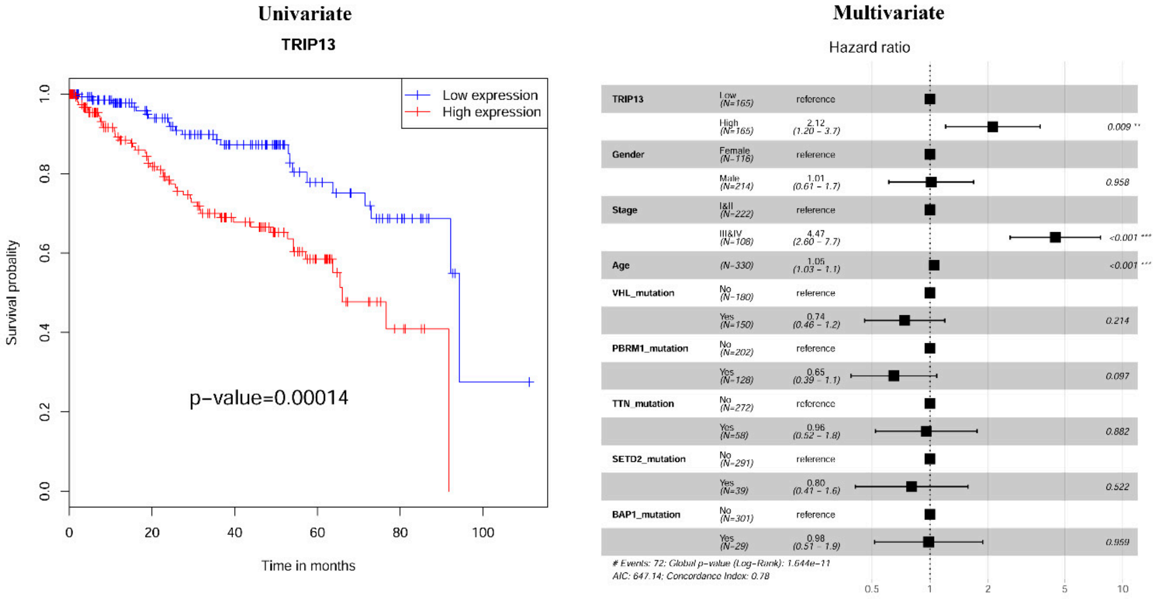

Figure S6. The expression of *TRIP13* associated with survival outcomes in KIRC. Kaplan-Meier curves showed that patients with high expression of *TRIP13* had worse outcome compared with those with low expression of *TRIP13*. Multivariate Cox regression analysis showed that *TRIP13* was an independent prognostic factor.

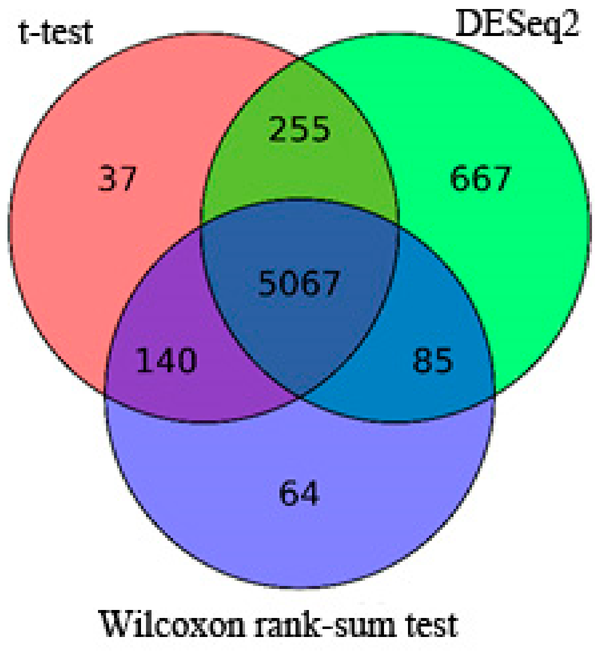

Figure S7. The venn diagram of DEGs found by Student's *t*-test, wilcoxon rank-sum test and DESeq2 under the same cut-off.

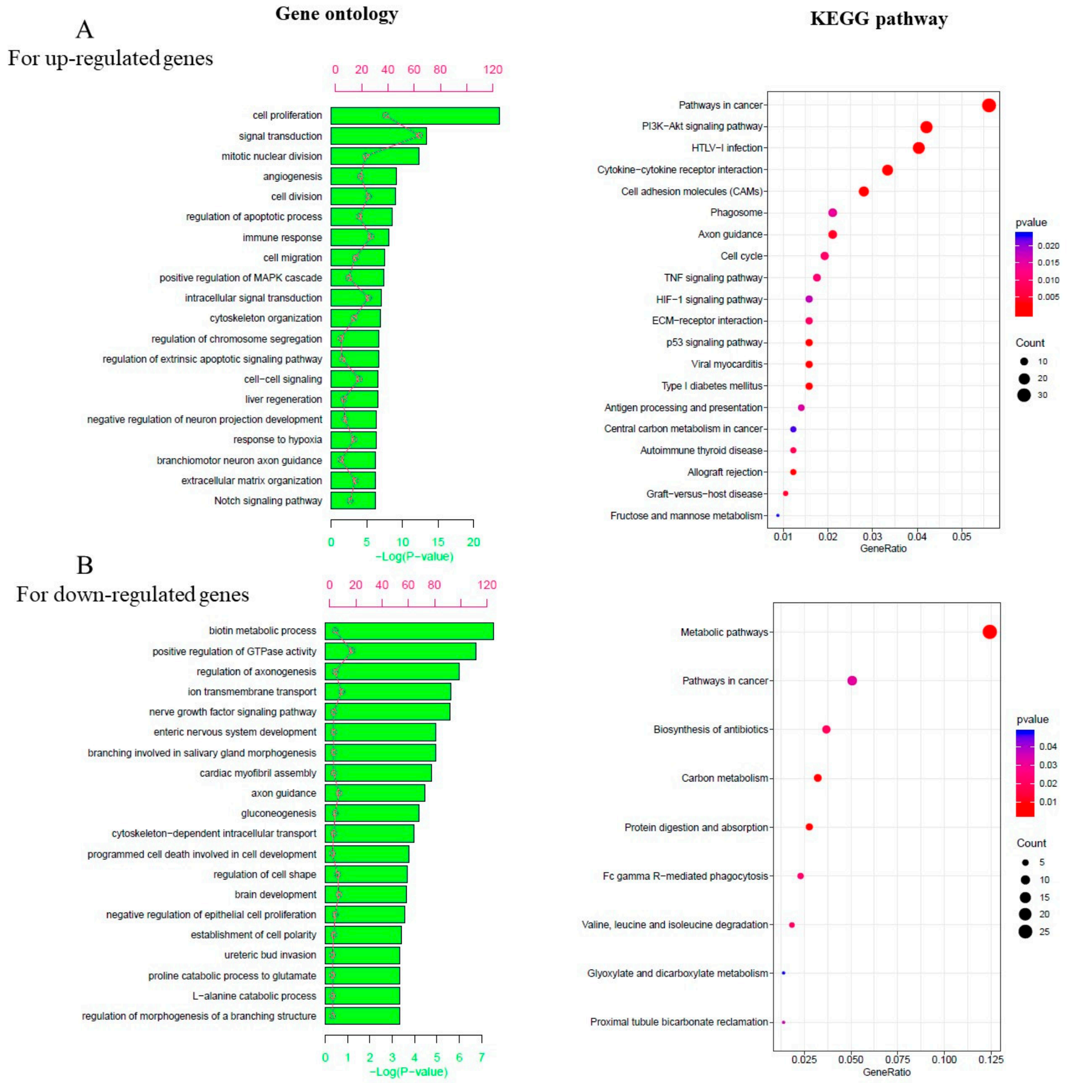

Figure S8. Enrichment analysis of MDEGs. The top 20 significant enriched Gene Ontology (left panel) and KEGG pathways (right panel) for (A) 629 up-regulated MDEGs; and for (B) 234 down-regulated MDEGs.

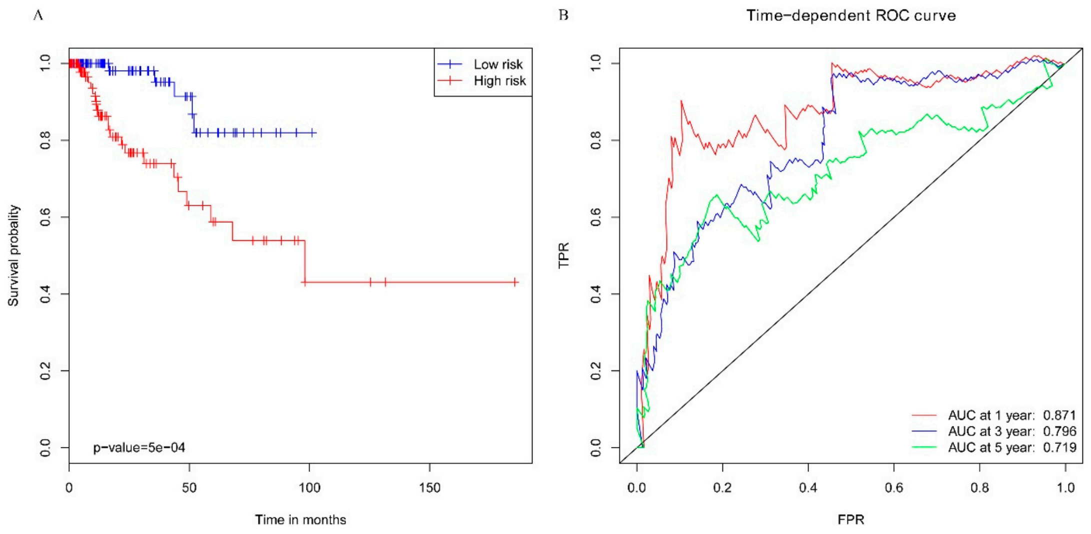

Figure S9. The survival analysis for KIRP. (A) The Kaplan–Meier plot (low-risk vs. high-risk group) for KIRP patients; (B) Receiver operating characteristic (ROC) analysis of the sensitivity and specificity of the survival time by a risk score based on the seven-MDEG signature of KIRP.
